# Interrater Reliability Estimation via Maximum Likelihood for Gwet’s Chance Agreement Model

**DOI:** 10.4236/ojs.2024.145021

**Published:** 2024-10-28

**Authors:** Alek M. Westover, Tara M. Westover, M. Brandon Westover

**Affiliations:** 1 Massachusetts Institute of Technology, Boston, MA, USA; 2 Harvard Medical School, Beth Israel Deaconess Medical Center, Boston, MA, USA

**Keywords:** Interrater Reliability, Agreement, Reliability, Kappa

## Abstract

Interrater reliability (IRR) statistics, like Cohen’s kappa, measure agreement between raters beyond what is expected by chance when classifying items into categories. While Cohen’s kappa has been widely used, it has several limitations, prompting development of Gwet’s agreement statistic, an alternative “kappa”statistic which models chance agreement via an “occasional guessing” model. However, we show that Gwet’s formula for estimating the proportion of agreement due to chance is itself biased for intermediate levels of agreement, despite overcoming limitations of Cohen’s kappa at high and low agreement levels. We derive a maximum likelihood estimator for the occasional guessing model that yields an unbiased estimator of the IRR, which we call the maximum likelihood kappa ($\kappa_{\text{ML}}$). The key result is that the chance agreement probability under the occasional guessing model is simply equal to the observed rate of disagreement between raters. The $\kappa_{\text{ML}}$ statistic provides a theoretically principled approach to quantifying IRR that addresses limitations of previous κ coefficients. Given the widespread use of IRR measures, having an unbiased estimator is important for reliable inference across domains where rater judgments are analyzed.

## 1. Introduction

Interrater reliability (IRR) (also known as “kappa” (κ)) statistics, are used to measure agreement between two raters or coders classifying items into mutually exclusive categories. κ statistics are widely used in fields such as psychology and medicine to evaluate the reliability or consistency of expert judgments [1].

Simply calculating the percentage of cases where raters agree does not account for the possibility that some agreement occurs by chance. κ is designed to measure the the degree of agreement between raters *beyond* what is expected by chance. Assume two raters independently classify N cases into categories + and −, and denote by $N_{a}$ the number of cases on which they agree. Assume $N_{c}$ agreements occur by chance, and the rest $N_{k}$ are due to knowledge (not due to chance), so that $N_{a}=N_{c}+N_{k}$. The number of cases remaining after subtracting chance agreements is $N-N_{c}$. Thus the percentage of the observed agreement $N_{a}$ in excess of chance agreement is:

$$\kappa =\frac{N_{a}-N_{c}}{N-N_{c}}=\frac{N_{k}}{N-N_{c}}=\frac{P_{a}-P_{c}}{1-P_{c}},$$

where $P_{a}=N_{a}/N$ denotes the observed percent agreement, and $P_{c}=N_{c}/N$ is the percent agreement due to chance. $P_{a}$ is observed, whereas $P_{c}$ must be estimated.

Several approaches have been proposed to estimate the probability of chance agreement. The approach used most commonly in the past (Cohen’s κ) has recently fallen under criticism [2] [3], leading to a new approach (Gwet’s κ) which has been gained popularity over the past several years [1] [4]-[7]. However, we show that the new approach is biased. We demonstrate an unbiased approach to estimating κ based on maximum likelihood estimation.

## 2. Cohen’s Kappa and Its Limitations

Historically, the most commonly used κ statistic has been Cohen’s κ [8] [9], which quantifies interrater reliability for two raters applying binary ratings. Other approaches are discussed at length in [10]-[12].

Cohen proposed calculating the probability of chance agreement $P_{c}$ based on an ‘always guess’ model. Suppose two raters A and B independently assign N items to two categories, + and −. Let the numbers of items assigned to each category be $N_{A}^{+},N_{A}^{-},N_{B}^{+},N_{B}^{-}$, and the number of items on which they agree be $N_{a}$. Now consider what percentage of cases raters A and B would be expected to agree on if they assigned the same numbers of items to each category as they do in the observed data, but made the assignments at random (“guessing”). Under this model, A and B classify items as + with probabilities $p_{A}^{+}=N_{A}^{+}/N,p_{B}^{+}=N_{B}^{+}/N$, and as – with probabilities $p_{A}^{-}=N_{A}^{-}/N,\;p_{B}^{-}=N_{B}^{-}/N$. Any agreements under this model occur by chance, with probability

$$P_{c}=p_{A}^{+}p_{B}^{+}+p_{A}^{-}p_{B}^{-}.$$

### Critiques of Cohen’s Model

Two main criticisms have been raised against Cohen’s κ. First, Cohen’s κ produces “paradoxical” results under certain circumstances [2] [10] [11] [13]: high levels of observed agreement can accompany a low κ value. This happens because Cohen’s κ depends only on the rates of ratings in the data. Thus, if raters A and B score most cases as class +, it may be because they correctly recognize that most cases are +, yet Cohen’s κ cannot give credit for agreement due to expertise. This problem is most pronounced when the proportion of classes in the data deviates from 50% [12].

Second, some authors [12] [14] dispute the idea that κ “takes into account” chance agreement. Truly doing this requires a realistic model of how chance affects rater decisions; Cohen’s ‘always guess’ model is unrealistic as a model of how raters behave. For this reason κ can be misleading in situations such as the diagnosis of rare diseases. In these scenarios, κ tends to underestimate agreement on the rare category [15]. κ is thus considered an overly conservative measure of agreement [16].

## 3. Gwet’s Kappa: An Improved Model of Chance Agreement

Gwet proposed an alternative to Cohen’s κ, which we call Gwet’s κ (also known as AC1 (Agreement Coefficient 1)) that addresses the limitations discussed above [12]. Gwet’s key contribution was a more realistic model of chance agreement, $P_{c}$, which we call the “occasional guessing” model. Because this model addresses the limitations of Cohen’s κ, Gwet’s κ has been increasingly adopted in studies of IRR [1] [4]-[7]. However, as we show below, Gwet’s κ also has important limitations. Specifically, the formula Gwet proposed for estimating κ is biased.

### 3.1. The “Occasional Guessing” Model for Chance Agreement

Gwet suggested that a more realistic model for how chance agreement occurs is:

1. Cases are easy or hard. Raters always classify easy cases correctly, and for hard cases, they guess with equal probability. Thus, for hard cases, the probability of agreement is 1/2.
2. The fraction of hard cases is r.

### 3.2. Theoretical Value of κ under the Occasional Guessing Model

Using this model, we can calculate the theoretical true value of κ, denoted $\kappa^{*}$. For any case evaluated by two raters consider the following events: A={Ratersagree}, and R={thecaseishard:ratersguessrandomly}. Then the probability of agreement due to chance (arising out of guessing) for any case is

$$P_{c}=P(A,R)=P(R)P(A|R)=r/2$$

The overall probability of agreement is

$$P_{a}=P\left(A\right)\;=P\left(A,R\right)+P\left(A,\bar{R}\right)\;=P\left(R\right)P\left(A|R\right)+P\left(\bar{R}\right)P\left(A|\bar{R}\right)\;=r/2+\left(1-r\right)\;=1-r/2.$$

Thus, the expected proportion of beyond-chance agreement is

$$\kappa^{*}=\frac{P_{a}-P_{c}}{1-P_{c}}=\frac{1-r}{1-r/2}.$$

We note that r can also be expressed in terms of κ, as

$$r=\frac{1-\kappa^{*}}{1-\kappa^{*}/2}.$$

It is easy to check that $0<\kappa <P_{a}$. Also, noting that κ=κ(r), we observe that for high and low values of r, we get κ(0)=1,κ(1)=0.

Any estimate of κ whose expected value deviates from the theoretical value $\kappa^{*}$ is said to be *biased*. We next consider Gwet’s proposal for estimating κ, and will show that it is biased in some important settings.

### 3.3. Gwet’s Formula for the Probability of Chance Agreement

Gwet proposed a formula for r=P(R) based on the following heuristic argument. Consider the random variable

$$X_{+}=\left\{\begin{array}{ll} 1 & \text{if}\;\text{a}\;\text{rater}\;\text{classifies}\;\text{a}\;\text{given}\;\text{case}\;\text{as}+\\ 0 & \text{otherwise} \end{array}\right.$$

The variance of $X_{+}$ is $\text{Var}\left(X_{+}\right)=\pi_{+}\left(1-\pi_{+}\right)$, where $\pi_{+}$ is the average rate at which raters assign cases to the “+” category. The maximum possible variance for classification is reached when rating is done completely at random, with each category assigned with probability 1/2, in which case the variance is ${\text{Var}}_{\text{max}}=1/2(1-1/2)=1/4$. Gwet suggested that a reasonable measure of the randomness with which raters choose the + category is the ratio of the observed choice variance to the maximal possible variance, *i.e*. $P(R)\approx \text{Var}\left(X_{+}\right)/V_{\text{max}}$, thus:

$$r=P(R)=\frac{\pi_{+}\left(1-\pi_{+}\right)}{1/2(1-1/2)}=4\pi_{+}\left(1-\pi_{+}\right),$$

This leads to chance agreement probability of

$$P_{c}=r/2=2\pi_{+}\left(1-\pi_{+}\right),$$

which can be substituted into $\kappa =\left(P_{a}-P_{c}\right)/\left(1-P_{c}\right)$.

### 3.4. Gwet’s κ Is Biased

Gwet showed that, when considered from the point of view of the ‘occasional guessing’ model of chance agreement, Cohen’s κ and several other well-known κ and κ-like statistics for interrater agreement are biased, particularly at high levels of agreement [1] [12]. By contrast, Gwet’s formula is accurate (nearly unbiased, i.e. $\kappa \approx \kappa^{*}$) when agreement between raters $P_{a}$ is high or low, overcoming a key limitation of Cohen’s κ [1] [12]. This is easy to show: When agreement is high, $P_{a}\approx 1$, we have $\kappa \approx \left(1-P_{c}\right)/\left(1-P_{c}\right)=1$, regardless of $P_{c}$. When agreement is low (both raters guessing all the time, r=1), agreement occurs in approximately half the cases, $P_{a}\approx 1/2$, approximately half of the ratings are positive, $\pi_{+}\approx 1/2$, and $P_{c}=2(1/2)(1-1/2)=1/2$, and κ=(1/2-1/2)/(1-1/2)=0.

However, for intermediate levels of agreement, Gwet’s formula is biased. We show this by expressing $\pi_{+}$ in terms of r, substituting into Gwet’s formula for $P_{c}$, then comparing this with the true value $P_{c}=r/2$. The proportion of + ratings is the sum of the proportions of + ratings on hard cases, r/2, and easy cases, (1-r)q, where q∈[0,1] is the proportion of easy cases whose true rating is +. Thus $\pi_{+}=r/2+(1-r)q$, and Gwet’s formula gives $P_{c}=2(r/2+(1-r)q)(1-r/2-(1-r))q$. The deviation of Gwet’s formula for $P_{c}$ from the true value r/2 is

$$\Delta P_{c}=2(r/2+(1-r)q)(1-r/2-(1-r)q)-r/2=r/2-r^{2}/2.$$

**Figure 1.**
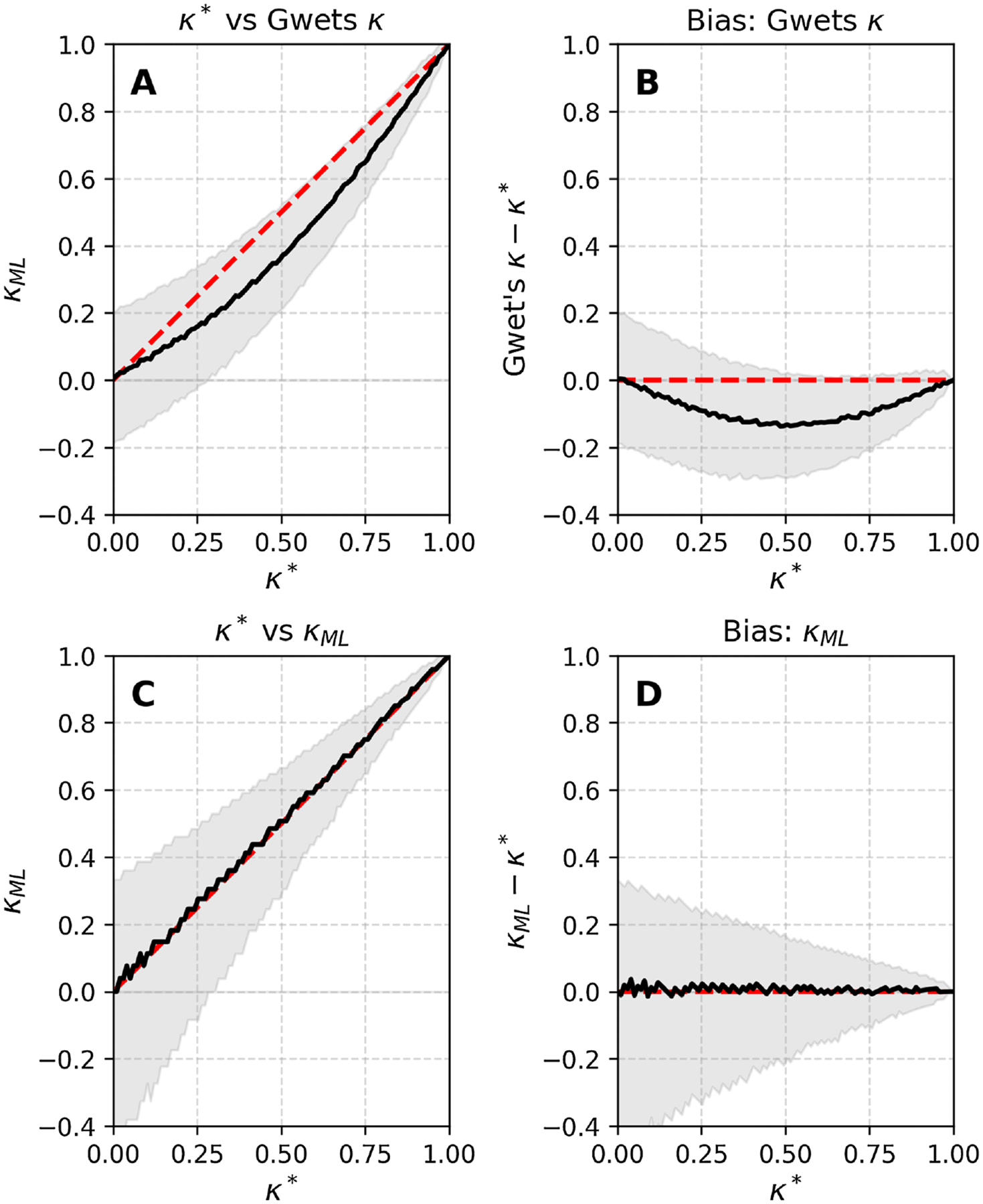
(A) True $\kappa =\kappa^{*}$ vs Gwet’s κ. (B) Bias (Gwet’s $\kappa -\kappa^{*}$). (C) $\kappa^{*}$ vs $\kappa_{\text{ML}}$. (D) $\text{Bias}\left(\kappa_{\text{ML}}-\kappa^{*}\right)$.

Note that this bias does not depend on q. Figure 1(A) & Figure 1(B) illustrate the bias and 95% confidence intervals for 2 raters scoring N=100 cases, where q=0.2, over the entire range of possible true values $\kappa^{*}$ of the underlying IRR.

## 4. Maximum Likelihood Estimation of P(R)

Here we present a direct approach to estimating P(R)=r in Gwet’s occasional guessing model. Unlike Gwet’s κ, the ML κ is not based on a heuristic approximation. Rather, we derive $\kappa_{\text{ML}}$ by writing down the likelihood of the observed data under the occasional guessing model and then solving for the r that maximizes that likelihood.

Let $X=\left[X_{1},X_{2},\cdots ,X_{N}\right]$ represent the agreement and disagreements for the N cases, where $X_{i}=0$ indicates disagreement and $X_{i}=1$ indicates agreement. When event R occurs (random guessing), we have $P\left(X_{i}=0|R\right)=P\left(X_{i}=1|R\right)=1/2$. For easy cases, raters are not guessing (i.e. $\overset{‾}{R}$ occurs), and we have $P\left(X_{i}=0|\overset{‾}{R}\right)=0,\;P\left(X_{i}=1|\overset{‾}{R}\right)=1$. The probability that raters guess is P(R)=r. The probabilities for $X_{i}$ conditional on r are

$$P\left(X_{i}=0|r\right)=P\left(R\right)P\left(X_{i}=0|R\right)+P\left(\bar{R}\right)P\left(X_{i}=0|\bar{R}\right)=r/2\;P\left(X_{i}=1|r\right)=P\left(R\right)P\left(X_{i}=1|R\right)+P\left(\bar{R}\right)P\left(X_{i}=1|\bar{R}\right)=1-r/2$$

The likelihood function for the data is: $P(X|r)=\prod_{i=1}^{N}P\left(X_{i}|r\right)$, so the log-likelihood is $L(X|r)=\sum_{i=1}^{N}\text{log}\;P\left(X_{i}|r\right)$. Splitting the sum into $N_{d}$ terms in which they disagree $\left(X_{i}=0\right)$ and $N_{a}$ terms in which they agree $\left(X_{i}=1\right)$, we get

$$L\left(X|r\right)=N_{d}\;\text{log}\;P\left(X=0|r\right)+N_{a}\;\text{log}\;P\left(X=1|r\right)\;=N_{d}\;\text{log}\frac{r}{2}+N_{a}\;\text{log}\left(1-r/2\right)$$

Taking the derivative of L(X∣r) with respect to r, setting it equal to zero, and solving, we get:

$$\frac{\partial }{\partial r}L\left(X,r\right)=N_{d}/r_{\text{ML}}-\frac{1}{2}N_{a}/\left(1-r_{\text{ML}}/2\right)=0\;\Rightarrow r_{\text{ML}}=\frac{2N_{d}}{N},$$

where $N=N_{d}+N_{a}$. Note that $N_{d}/N=P_{d}$ the probability of disagreement.

This result makes sense: Given that the probability of agreement when raters guess is 1/2, the best estimate from the data of the number of times at least one rater was in fact guessing is twice the number of observed disagreements.

From the above calculation it follows that the estimated probability of agreement due to chance is

$$P_{c}=P(R)P(A|R)=r_{\text{ML}}/2=N_{d}/N.$$

### $\kappa_{ML}$ Is Unbiased

We now show that the expected value of the ML estimator for κ is equal to the theoretical value, hence $\kappa_{\text{ML}}$ is an unbiased estimator of $\kappa^{*}$.

Recall that $r_{\text{ML}}=\left(2N_{d}\right)/N$ is the probability of chance agreement used in calculating $\kappa_{\text{ML}}$, where $N_{d}$ is the number of disagreements observed between the two raters performing binary assignments. We can rewrite this as $N_{d}=E\left[\sum_{i=1}^{N}\bar{X_{i}}\right]$, since $X_{i}=0$ denotes disagreements, and $X_{i}=1$ in cases of agreement. Thus,

$$\text{E}\left[r_{\text{ML}}\right]=\text{E}\left[2N_{d}/N\right]\;=\frac{2}{N}\text{E}\left[\sum_{i=1}^{N}\bar{X_{i}}\right]\;=\frac{2}{N}N\cdot \left(r/2\right)\;=r$$

Consequently,

$$E\left[\kappa_{\text{ML}}\right]=\frac{1-E\left[r_{\text{ML}}\right]}{1-E\left[r_{\text{ML}}\right]/2}=\frac{1-r}{1-r/2}=\kappa^{*}$$

Figure 1(C) & Figure 1(D) illustrate the estimation of $\kappa_{\text{ML}}$ in a case with N=100 cases scored by 2 raters, including bootstrap estimates of the 95% confidence intervals.

## 5. Variance of $\kappa_{ML}$

We now compute the variance of our estimate of $r_{\text{ML}}$. The key computation is computing the second moment of $N_{d}$.

$$\text{E}\left[N_{d}^{2}\right]=\text{E}\left[{\left(\sum_{i}\bar{X_{i}}\right)}^{2}\right]\;=\sum_{i\neq j}P\left(\bar{X_{i}}\bar{X_{j}}\right)+\sum_{i}P\left(\bar{X_{i}}\right)\;=\left(N^{2}-N\right)r^{2}/4+Nr/2.$$

Thus,

$$\text{Var}\left[r_{\text{ML}}\right]=\frac{4}{N^{2}}\left(\text{E}\left[N_{d}^{2}\right]-\text{E}{\left[N_{d}\right]}^{2}\right)\;=\frac{4}{N^{2}}\left(\left(N^{2}-N\right)r^{2}/4+Nr/2-{\left(Nr/2\right)}^{2}\right)\;=\frac{r\left(2-r\right)}{N}.$$

Let $f(r)=\frac{1-r}{1-r/2}$. The maximum derivative of f over r∈[0,1] is 2. Thus, we have for all ϵ>0,r∈[ϵ,1]:

$$\left|f\left(r\right)-f\left(r-\epsilon \right)\right|\leq 2\epsilon .$$

In other words, a confidence interval for $r\in \left[r_{0}-\delta ,r_{0}+\delta \right]$ translates into a confidence interval for $\kappa_{\text{ML}}$ which is $\kappa_{\text{ML}}\in \left[f\left(r_{0}\right)-2\delta ,f\left(r_{0}\right)+2\delta \right]$. Confidence intervals can also be calculated numerically using bootstrapping, as shown in Figure 1.

## 6. Multiple Categories

The preceding sections have dealt with the case of classifying into 2 categories. We can analogously derive $\kappa_{\text{ML}}$ and r in the case where there is instead an arbitrary number, n, of classes. To do this, we generalize the “occasional guessing” model so that, for hard cases, raters guess all n classes with equal probability. Under this model, the probability of agreement by guessing is

$$P_{c}=P(A,R)=P(R)P(A|R)=r/n,$$

and the overall probability of agreement is

$$P_{a}=P\left(A,R\right)+P\left(A,\bar{R}\right)\;=P\left(R\right)P\left(A|R\right)+P\left(\bar{R}\right)P\left(A|\bar{R}\right)\;=r/n+\left(1-r\right)\cdot 1\;=1+\frac{r}{n}\left(1-n\right).$$

Now, to find the theoretical $\kappa^{*}$ in terms of r,

$$\kappa^{\text{*}}=\frac{P_{a}-P_{c}}{1-P_{c}}\;=\frac{1+\frac{r}{n}\left(1-n\right)-r/n}{1-r/n}\;=\frac{1-r}{1-r/n}.$$

Next we derive the ML estimator of r. Let $X=\left[X_{1},X_{2},\cdots ,X_{N}\right]$ represent the agreement and disagreements for the N cases, where $X_{i}=0$ indicates disagreement and $X_{i}=1$ indicates agreement. When event R occurs (random guessing), we have $P\left(X_{i}=0|R,r\right)=P\left(X_{i}=1|R,r\right)=1/2$. When neither rater guesses (*i.e*. event $\overset{‾}{R}$ occurs), we have $P\left(X_{i}=0|\overset{‾}{R},r\right)=0,\;P\left(X_{i}=1|\overset{‾}{R},r\right)=1$. The probability that raters guess randomly is P(R)=r. The probabilities for $X_{i}$ conditional on r are

$$P\left(X_{i}=0|r\right)=1-P_{a}=\frac{r}{n}\left(n-1\right)\;P\left(X_{i}=1|r\right)=P_{a}=1+\frac{r}{n}\left(1-n\right)$$

Now, to find $\kappa_{\text{ML}}$, we maximize the likelihood function for the data $P(X|r)=\prod_{i=1}^{N}P\left(X_{i}|r\right)$, or the log-likelihood $L(X|r)=\sum_{i=1}^{N}\text{log}\;P\left(X_{i}|r\right)$. Splitting the sum into $N_{d}$ terms with $X_{i}=0$ and $N_{a}$ terms with $X_{i}=1$, we get

$$L\left(X|r\right)=N_{d}\;\text{log}\;P\left(X=0|r\right)+N_{a}\;\text{log}\;P\left(X=1|r\right)\;=N_{d}\;\text{log}\left(\frac{r}{n}\left(n-1\right)\right)+N_{a}\;\text{log}\left(1+\frac{r}{n}\left(1-n\right)\right).$$

Taking the derivative with respect to r, setting it equal to zero, and solving, we get:

$$\frac{\partial }{\partial r}L\left(X,r\right)=N_{d}\cdot \frac{n}{r\left(n-1\right)}\cdot \frac{n-1}{n}+N_{a}\frac{1}{1+\frac{r}{n}\left(1-n\right)}\cdot \frac{n}{1-n}\;=\frac{N_{d}}{r}+\frac{N_{a}}{\frac{n}{1-n}+r}=0\;\Rightarrow r_{\text{ML}}=\frac{N_{d}}{N}\frac{n}{n-1}$$

where $N=N_{d}+N_{a}$.

## 7. Conclusions

We have presented a maximum likelihood approach to estimating the chance agreement probability $P_{c}$ in Gwet’s “occasional guessing” model of interrater agreement. Our estimator, $\kappa_{\text{ML}}$, is derived directly from the likelihood function of the data under this model, rather than relying on heuristic approximations as in Gwet’s κ.

We have shown that the maximum likelihood estimator $r_{\text{ML}}$ for the probability of guessing r is simply twice the observed disagreement rate between raters. Consequently, the chance agreement probability estimate $P_{c}$ used in $\kappa_{\text{ML}}$ is the observed disagreement rate. We have also generalized this result to the case of raters scoring cases that can belong to multiple classes.

A key advantage of $\kappa_{\text{ML}}$ is that it is an unbiased estimator of the true value of κ predicted by the occasional guessing model. In contrast, we have demonstrated that Gwet’s formula for $P_{c}$, while overcoming certain limitations of Cohen’s κ, is itself biased for intermediate levels of agreement.

We have also provided the variance of the $\kappa_{\text{ML}}$ estimator, which can be used to construct confidence intervals. The variance depends on both the true value of r and the sample size N, decreasing as N increases as expected for a consistent estimator.

In summary, $\kappa_{\text{ML}}$ provides a principled approach to estimating chance agreement in the occasional guessing model, addressing limitations of previous κ statistics. As the use of interrater reliability measures continues to grow across fields, having an unbiased estimator is important for obtaining reliable inferences from data.

## Data Availability

The code that supports the findings of this study is available from the corresponding author upon request.

## Abbreviations

The following abbreviations are used in this manuscript:

IRR: Interrater reliability
ML: Maximum likelihood
AC1: Agreement coefficient 1
